# Genome-Wide Analysis in Human Colorectal Cancer Cells Reveals Ischemia-Mediated Expression of Motility Genes via DNA Hypomethylation

**DOI:** 10.1371/journal.pone.0103243

**Published:** 2014-07-31

**Authors:** Karolina Skowronki, Joseph Andrews, David I. Rodenhiser, Brenda L. Coomber

**Affiliations:** 1 Department of Biomedical Sciences; Ontario Veterinary College; University of Guelph; Guelph, ON, Canada; 2 Departments of Biochemistry, Oncology and Paediatrics; University of Western Ontario; London Regional Cancer Centre and Children’s Health Research Institute; London, ON, Canada; Howard University, United States of America

## Abstract

DNA hypomethylation is an important epigenetic modification found to occur in many different cancer types, leading to the upregulation of previously silenced genes and loss of genomic stability. We previously demonstrated that hypoxia and hypoglycaemia (ischemia), two common micro-environmental changes in solid tumours, decrease DNA methylation through the downregulation of DNMTs in human colorectal cancer cells. Here, we utilized a genome-wide cross-platform approach to identify genes hypomethylated and upregulated by ischemia. Following exposure to hypoxia or hypoglycaemia, methylated DNA from human colorectal cancer cells (HCT116) was immunoprecipitated and analysed with an Affymetrix promoter array. Additionally, RNA was isolated and analysed in parallel with an Affymetrix expression array. Ingenuity pathway analysis software revealed that a significant proportion of the genes hypomethylated and upregulated were involved in cellular movement, including *PLAUR* and *CYR61*. A Matrigel invasion assay revealed that indeed HCT116 cells grown in hypoxic or hypoglycaemic conditions have increased mobility capabilities. Confirmation of upregulated expression of cellular movement genes was performed with qPCR. The correlation between ischemia and metastasis is well established in cancer progression, but the molecular mechanisms responsible for this common observation have not been clearly identified. Our novel data suggests that hypoxia and hypoglycaemia may be driving changes in DNA methylation through downregulation of DNMTs. This is the first report to our knowledge that provides an explanation for the increased metastatic potential seen in ischemic cells; *i.e.* that ischemia could be driving DNA hypomethylation and increasing expression of cellular movement genes.

## Introduction

Solid tumours undergo a fundamental process known as angiogenesis (recruitment of neo-vasculature) in order to maintain adequate levels of oxygen and nutrients to the expanding mass [1]. Tumour blood vessels are highly abnormal and due to the rate of tumour growth which exceeds the rate of angiogenesis, areas of a tumour will develop reduced blood flow, or ischemia [2]. In addition to other changes, ischemic regions will have areas of hypoxia and hypoglycaemia, and while the impact of hypoglycaemia on tumorigenesis is not well studied, the role of hypoxia in cancer progression is well documented and includes increasing genetic instability, and stimulating cell invasion and angiogenesis, thereby contributing to metastasis [3].

Cellular movement/migration is a critical step in metastasis, which is the cause of most cancer-related mortalities [4]. A variety of molecular changes can cause a cell to be motile and travel into the lymphatic or vasculature system, eventually leading to arrest at a new site in the body [5]. Cells within an ischemic environment have a higher metastatic potential through the upregulation of genes such as *VEGF* to stimulate angiogenesis, and *urokinase plasminogen activator* (*uPA*) which activates plasmin to degrade the ECM [5]. Through stabilization of a subunit of the hypoxia-responsive transcription factor, HIF1α, hypoxia drives expression of these two genes, and many others, promoting metastasis. Cells may also be rendered more motile via epigenetic reprogramming. Several relevant genes such as *uPA* [6] and *S100A4* [7] have altered epigenetic patterning in cancer cells, allowing cell morphology to be modified to a more metastatic-favourable state. What remains unknown is if epigenetic modifications and ischemia are linked in promoting tumour metastasis.

DNA methylation is an important epigenetic mechanism which regulates gene expression [8]. Methylated DNA is associated with transcriptional silencing, since the methyl groups at cytosine residues change the configuration of DNA and therefore prevent transcription factors from binding [9], as well as triggering the recruitment of repressors and chromatin modifying enzymes that further silence expression through chromatin condensation [10], [11]. Several DNA methyltransferases (DNMT) catalyze the conversion of cytosine to 5-methylcytosine [12], with the most commonly studied DNMTs being DNMT1 (a maintenance methyltransferase) and DNMT3a and DNMT3b (*de novo* methyltransferases) [13].

In cancer, two common disruptions to DNA methylation patterns are observed: promoter-specific hypermethylation and global hypomethylation [14]. Most studies have focused on DNA hypermethylation and the impact that silencing tumour suppressor genes has on tumour initiation, progression, and prognosis. DNA hypomethylation on the other hand has been overshadowed by the extensive attention to DNA hypermethylation in cancer therapy, even though hypomethylation was the first epigenetic disruption noted in cancer [15], [16]. Despite the initial lack of understanding regarding the importance of DNA hypomethylation in cancer, it is now known that repetitive sequences become demethylated and lead to genomic chaos [17], [18]. As with DNA hypermethylation, hypomethylation can be site-specific, thereby contributing to enhanced expression [16]. In addition to such gene-specific effects, genomic instability, loss of imprinting [19] and abnormal X-chromosome activation also contribute to tumorigenesis [20].

Hoffmann and Schultz [16] hypothesized that DNA hypomethylation accelerates the adaptation of cancer cells to the dynamic microenvironment through selection for particular gene functions, such as motility. Evidence for this theory lies in the urokinase plasminogen activator *(uPA*) gene. A serine protease involved in degradation of the extracellular matrix, *uPA* has been shown to have a hypomethylated promoter region, leading to over-expression in both breast and prostate cancer and is directly associated with increased invasive and metastatic potential [6]. *Melanoma-associated antigen* (*MAGE*) is another of the group of genes found to be demethylated and upregulated in many cancers like melanoma, colorectal, gastric, and non-small cell lung cancer [21], [22], [23], [24], [25]. Overall, multiple mechanisms may be responsible for the dysregulation of DNA methylation in cancer. Overexpression of DNMTs has been reported in many different cancers [26] and explains aberrant hypermethylation, but less focus has gone into understanding DNA hypomethylation, even though DNA hypomethylation plays an equally important role in modifying the cancer epigenome.

Our previous work described a novel relationship between ischemia and DNA methylation, with reduced methyl cytosine seen in cells from ischemic regions of tumours [27]. We also found that in HCT116 human colorectal cancer cells, DNMT1, DNMT3a, and DNMT3b expression and activities were reduced by *in vitro* hypoxia and hypoglycaemia [28]. These changes in expression and activity were concurrent with hypomethylation of the *p16^INK4a^* promoter region in hypoglycaemic conditions [28]. In the present study, we explored the expression and methylation changes in HCT116 cells on a genome-wide level, utilizing a cross-platform approach to identify genes regulated by ischemic conditions. Given our previous findings, combined with the common observation of increased metastatic potential in ischemic tumours, we hypothesized that increased cellular motility of human colorectal cancer cells in ischemic conditions is due to the upregulation of motility-associated genes, and that these changes in expression are driven by ischemia-mediated DNA hypomethylation.

## Results

### Minimal copy number variation

**Figure 1:**
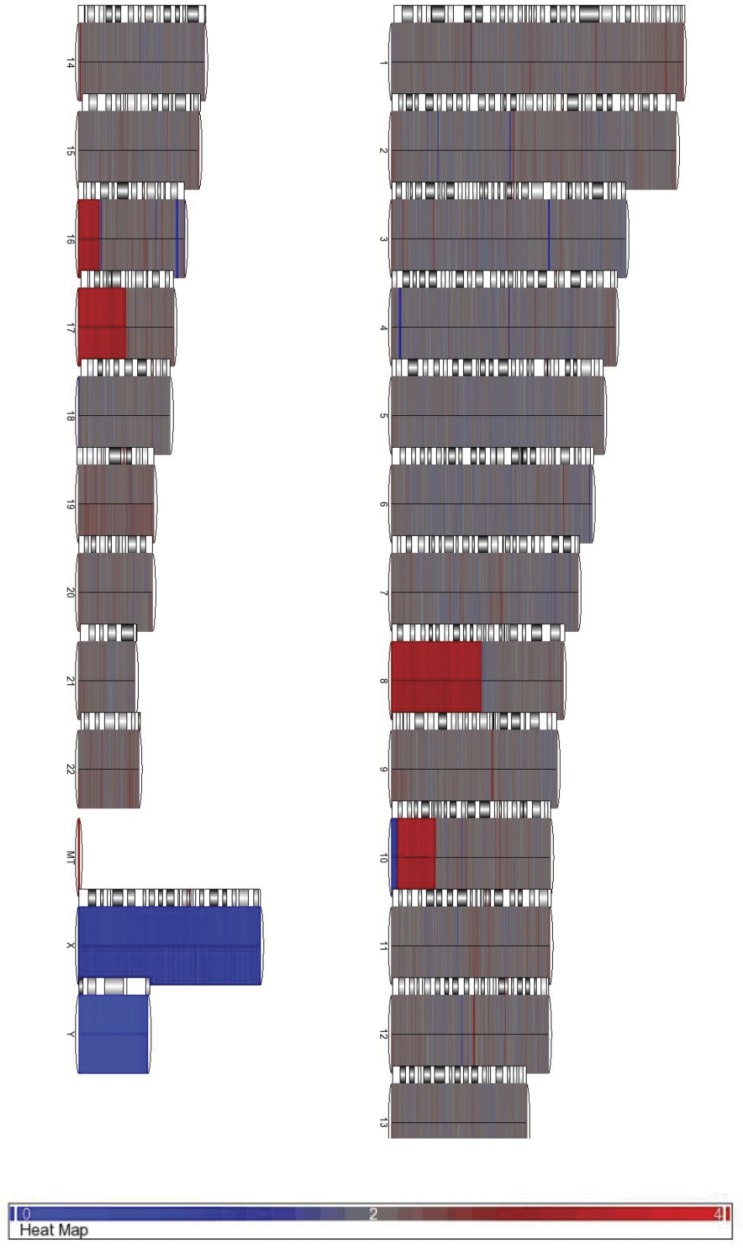
SNP 6.0 array of HCT116 cells to visualize copy number variation. Numbers (and X/Y) correspond to chromosomes, and MT represents mitochondrial DNA. Results were compared to a dataset from Affymetrix containing 270 mixed population samples from the International HapMap Project [29]. Regions in grey indicate no change in copy number, red indicates a copy number gain, and blue a copy number loss. This array was performed in duplicate.

The copy number of genes in the HCT116 cells was assessed with the Affymetrix SNP 6.0 array to determine if, and to what extent, the cells we used had drifted/mutated in culture compared to the original karyotype of this cell line. When compared to a pooled normal human karyotype from the International HapMap project database [29], the cells lacked extensive chromosomal abnormalities (Figure 1). We did observe regions on 8q, 10q, 16q, and 17q that displayed regional chromosomal gains (indicated in red), as previously reported by others [30]. Very few complete loss of copies were seen (blue), except for the Y chromosome which has been reported to be absent in 50–100% of HCT116 cells [31]. Thus, in comparison with other cancer cell lines, HCT116 have a relatively normal and stable karyotype with minimal chromosomal instability [30].

### Hypoglycaemia impacts more genes than hypoxia

Expression analysis was performed to quantitatively evaluate gene expression changes in HCT116 cells under ischemic conditions. The expression arrays indicated that following exposure to hypoxia for 48 hours, HCT116 cells had 310 and 1081 genes up- and down-regulated, respectively. When cells were exposed to hypoglycaemia, 1052 and 2433 genes were up- and down-regulated, respectively (Table 1). Analysis of promoter methylation using Affymetrix 1.0R tiling array revealed that hypoxia resulted in 1386 hypomethylated genes, and 1655 hypermethylated genes. Growth in hypoglycaemic conditions resulted in 1940 hypomethylated genes, and 1980 hypermethylated genes (Table 1). Thus, both expression and promoter methylation patterns were most heavily impacted by hypoglycaemia, as measured by total number of significantly changed genes.

**Table 1:** Summary of expression and promoter methylation arrays.

	Hypoxia		Hyperglycaemia	
Array Type	Change	Number of genes	Change	Number of genes
Expression	Upregulated	310	Upregulated	1052
	Downregulated	1081	Downregulated	2433
Promoter Methylation	Hypomethylated	1386	Hypomethylated	1940
	Hypermethylated	1655	Hypermethylated	1980

Total number of significantly changed genes in both expression (p<0.05) and promoter methylation (p<0.01) arrays, by hypoxia (A) or hypoglycaemia (B), compared to control.

### Cross-platform analysis

**Figure 2:**
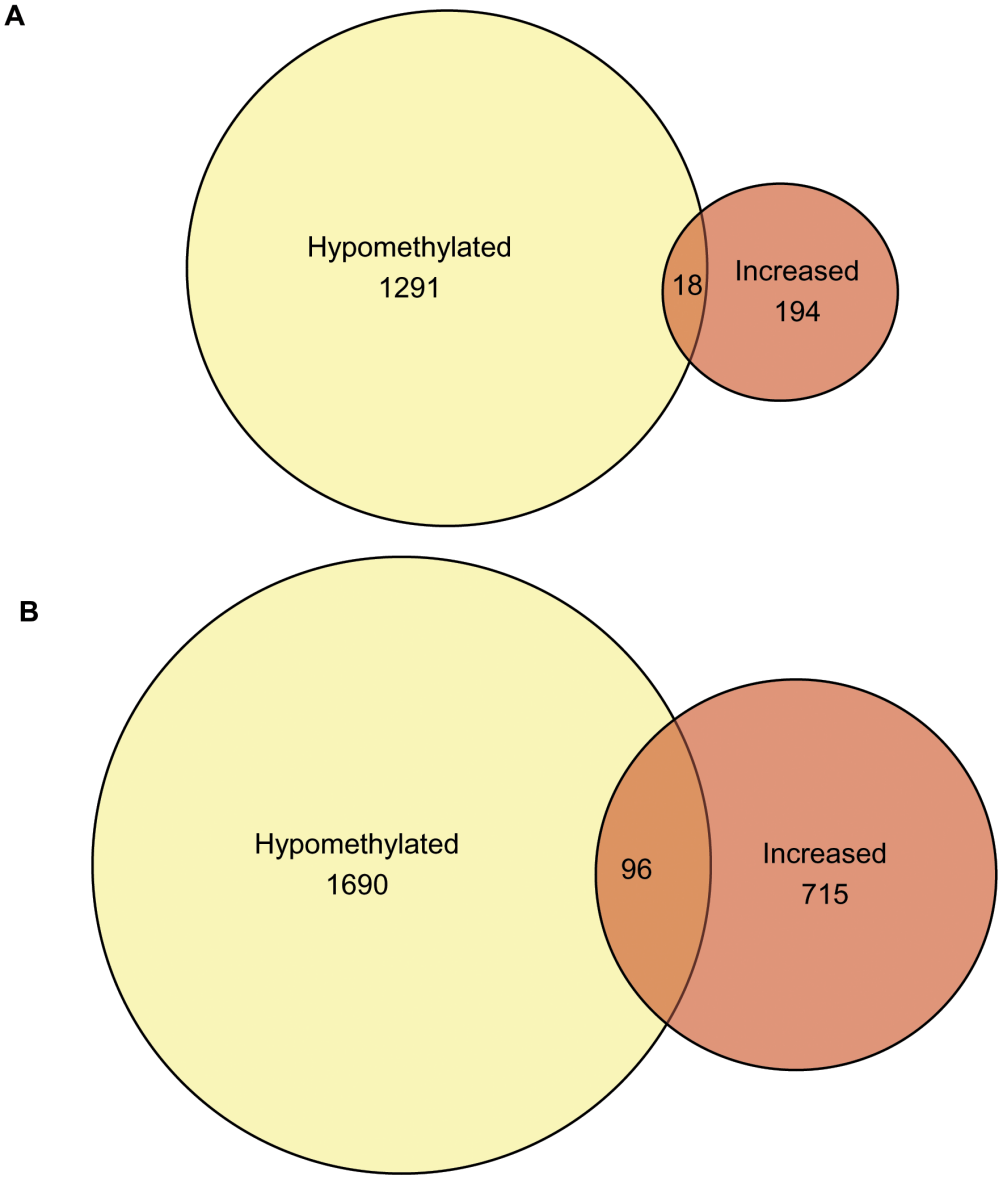
Venn diagram of significantly changed unique genes from cross-platform analysis. Number of genes which were both significantly hypomethylated and upregulated by hypoxia (A) and hypoglycaemia (B) are indicated in the overlapped regions.

Next, we assessed the functional significance of these complex gene expression and epigenetic (DNA methylation) profiles. Using the Partek Software Suite, unique genes that were hypomethylated and upregulated (as well as hypermethylated and downregulated) were overlaid in order to identify changes in gene expression associated with changes in promoter methylation. There were 58 unique genes hypermethylated and downregulated by hypoxic conditions, and 161 genes by hypoglycaemia (Data not shown). We focused on the genes that were hypomethylated and increased in expression, since our previous work showed DNMTs were repressed in ischemia. We observed that 18 and 96 unique genes were hypomethylated and upregulated by hypoxia and hypoglycaemia, respectively (Figure 2). Partial lists of these genes can be seen in Tables 2 and 3, with the complete list presented in Table I in Information S1.

**Table 2:** List of genes that were both hypomethylated and upregulated by hypoxia.

	Expression		Promoter Methylation	
Gene Symbol	p-value	Fold-Change	p-value	MAT score
ANKRD37	0.0003	25.14	0.008	−3.85
TMEM158	0.0022	13.21	0.007	−3.97
DDIT4	0.0001	8.51	0.010	−3.71
ALDOA	0.0050	5.26	0.010	−3.74
KDM3A	0.0031	5.01	0.009	−3.75
LDLR	0.0427	3.13	0.005	−4.24
SEMA4B	0.0006	2.91	0.009	−3.82
SPRY1	0.0260	2.90	0.009	−3.83
RBPJ	0.0061	2.81	0.007	−3.93
UFM1	0.0159	2.60	0.006	−4.03
PLAUR	0.0011	2.58	0.010	−3.72
LAMB3	0.0171	2.50	0.009	−3.76
NEDD4L	0.0004	2.45	0.007	−3.94
SCAI	0.0261	2.35	0.007	−4.00
PFKP	0.0025	2.27	0.009	−3.78
RYBP	0.0091	2.13	0.004	−4.34
CUL4B	0.0077	2.13	0.007	−3.92
AFAP1L1	0.0306	2.07	0.006	−4.12

Partek software was used to overlay the significant gene lists from the promoter methylation and expression arrays (the 18 genes from Figure 2A).

**Table 3:** List of the top 30 genes that were both hypomethylated and upregulated by hypoglycaemia.

	Expression		Promoter Methylation		
Gene Symbol	p-value	Fold-Change	p-value	MAT score	
CYR61	0.0000	31.35	0.009		−3.85
ETS1	0.0005	30.72	0.010		−3.77
KLF4	0.0002	22.29	0.009		−3.85
ELL2	0.0001	11.06	0.002		−5.10
NEDD4L	0.0001	11.03	0.002		−5.05
LATS2	0.0000	9.27	0.006		−4.13
SGMS1	0.0004	8.28	0.002		−5.29
INTS6	0.0008	8.22	0.007		−3.97
HEY1	0.0002	8.18	0.009		−3.82
COL12A1	0.0012	6.87	0.006		−4.14
PHIP	0.0006	5.61	0.009		−3.83
ARHGEF12	0.0003	5.54	0.004		−4.55
EPC1	0.0002	5.47	0.006		−4.12
SETX	0.0009	5.42	0.003		−4.64
C16orf52	0.0003	5.10	0.008		−3.93
PRDM10	0.0012	4.97	0.006		−4.15
DCAF10	0.0001	4.88	0.008		−3.94
NAB1	0.0021	4.80	0.003		−4.62
RYBP	0.0009	4.53	0.004		−4.40
TMOD3	0.0017	4.27	0.003		−4.58
TJP1	0.0013	4.23	0.009		−3.82
UBQLN1	0.0020	4.19	0.008		−3.95
OSBPL3	0.0001	4.12	0.005		−4.32
NRAS	0.0030	4.04	0.009		−3.83
GPR87	0.0000	3.91	0.009		−3.79
ESYT2	0.0002	3.68	0.010		−3.77
KLF3	0.0004	3.62	0.009		−3.79
SOCS5	0.0002	3.61	0.009		−3.83
GPBP1	0.0009	3.61	0.008		−3.94
SLC38A1	0.0006	3.55	0.004		−4.44

Partek software was used to overlay the significant gene lists from the promoter methylation and expression arrays (the 96 genes from Figure 2B).

### Ingenuity Pathway Analysis of hypomethylated and upregulated genes

Ingenuity Pathway Analysis (IPA) was used to analyze and classify the hypomethylated and upregulated genes into functional pathways, in order to better understand the biological relevance of the genes from our data set. The software determines if a biological function is enriched for in a data set by examining the whole gene list for functional annotations found in the ingenuity knowledge base, and performing a Fisher’s exact test comparing the ratio of genes with a given annotation in the data set against the ratio of genes having that same annotation in the whole population (*i.e.* all the genes on the HG U133 Plus 2.0 array). If the first ratio is significantly higher than the second, then the list is said to be enriched for genes having that annotation.

**Figure 3:**
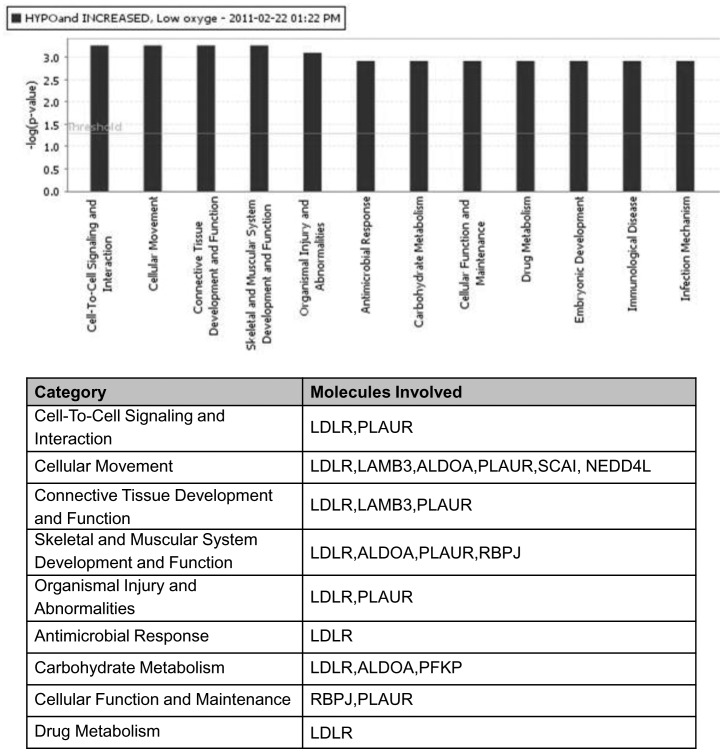
Functional analysis of genes hypomethylated and upregulated by hypoxia. Ingenuity Pathways Analysis database was used to assign genes to biological functions and determine functions that were enriched, based on statistical significance.

**Figure 4:**
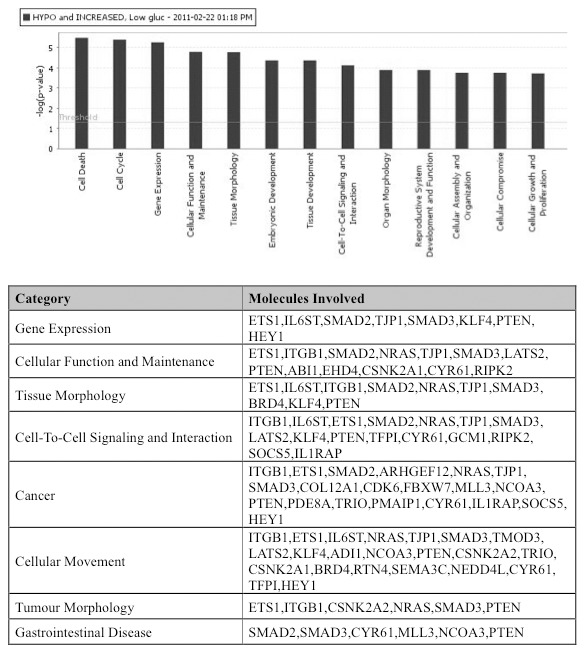
Functional analysis of genes hypomethylated and upregulated by hypoglycaemia. Ingenuity Pathways Analysis database was used to assign genes to biological functions and determine functions that were enriched, based on statistical significance.

**Figure 5:**
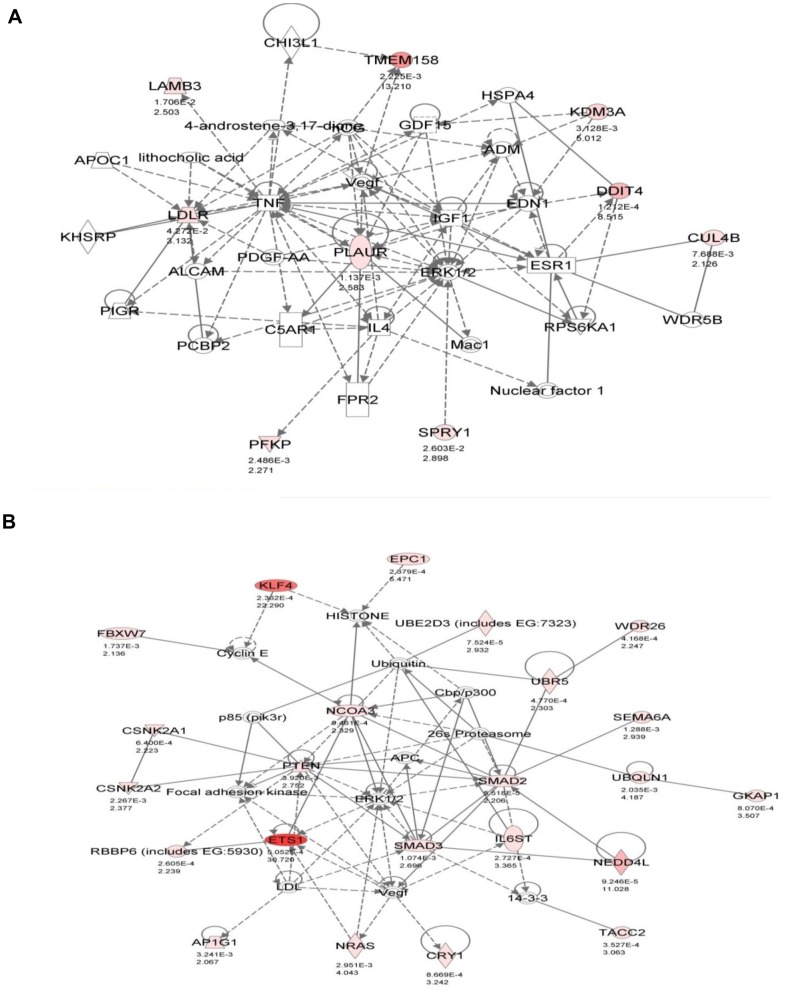
Top networks determined by Ingenuity Pathway Analysis. The top networks (most connections to central molecule) for hypomethylated and upregulated genes are shown for both hypoxia (A) and hypoglycaemia (B). The intensity of the red colouring of the molecules reflects the expression level as found in expression array (darker signifies greater increase in expression as compared to control conditions).

With hypoxia exposure, the top three cellular functions from our list of hypomethylated/upregulated genes were: cell-to-cell signaling, cellular movement, and connective tissue development (Figure 3). Hypoglycaemia enriched for changes in cell death, cell cycle, and regulation of gene expression (Figure 4). Although not within the top few functional categories, cellular movement was also an enriched function in the hypoglycaemic group. Network analysis was also performed to provide a graphical representation of known biological relationships of the hypomethylated and upregulated genes. The networks are generated by determining a focus molecule, and then examining the gene lists for the most connections to the focus molecule, based upon the literature in Ingenuity’s database. The top networks (having the most connections) for both hypoxia and hypoglycaemia involved genes which have roles in cellular movement. In hypoxic conditions, *PLAUR, LDLR*, and *LAMB3* (Figure 5A) were all in one network and have all been shown to be involved in cellular movement. The top network in hypoglycaemic conditions included cellular movement genes *ETS1, KLF4, IL6ST, NEDD4L*, and *NRAS* (Figure 5B). It is important to note that some genes in our list may have biological functions/annotations that the Ingenuity database does not recognize. As an example, *AFAP1L1* was not in the Ingenuity list of genes involved in cellular movement, however there is evidence for this molecule’s role in migration and invasion [32]. Based on the IPA analysis of functional pathways and networks, the category “cellular movement” appeared interesting and relevant and we further pursued the genes associated with these functions for array validation.

### Migration-associated genes are enriched in hypoxia and hypoglycaemia

The cross-platform analysis of genes hypomethylated and upregulated by hypoxia and hypoglycaemia revealed a number of genes that are involved in cellular movement: *LDLR, LAMB3, ALDOA, PLAUR, SCAI,* and *NEDD4L* in the hypoxic group (Figure 3), and *ITGB1, ETS1, IL6ST, NRAS, TJP1, SMAD3, TMOD3, LATS2, KLF4, ADI1, NCOA3, PTEN, CSNK2A2, TRIO, CSNK2A1, BRD4, RTN4, SEMA3C, NEDD4L*, *CYR61, TFPI,* and HEY1 in the hypoglycaemic group (Figure 4). In addition to these genes, we observed two others that were hypomethylated and upregulated likely also involved in cellular movement. *ANKRD37* and *AFAP1L1* in the ‘hypoxic group’ have been shown to be involved in migration and invasion of cancer cells (Table 2). Many of the “cellular movement” genes in our analysis are involved in mediating cellular attachment, either to other cells or the ECM, or in ECM degradation, all processes required for a cell to move within tissue and to enter blood vessels.

### Confirmation of increased expression of cellular movement genes

**Figure 6:**
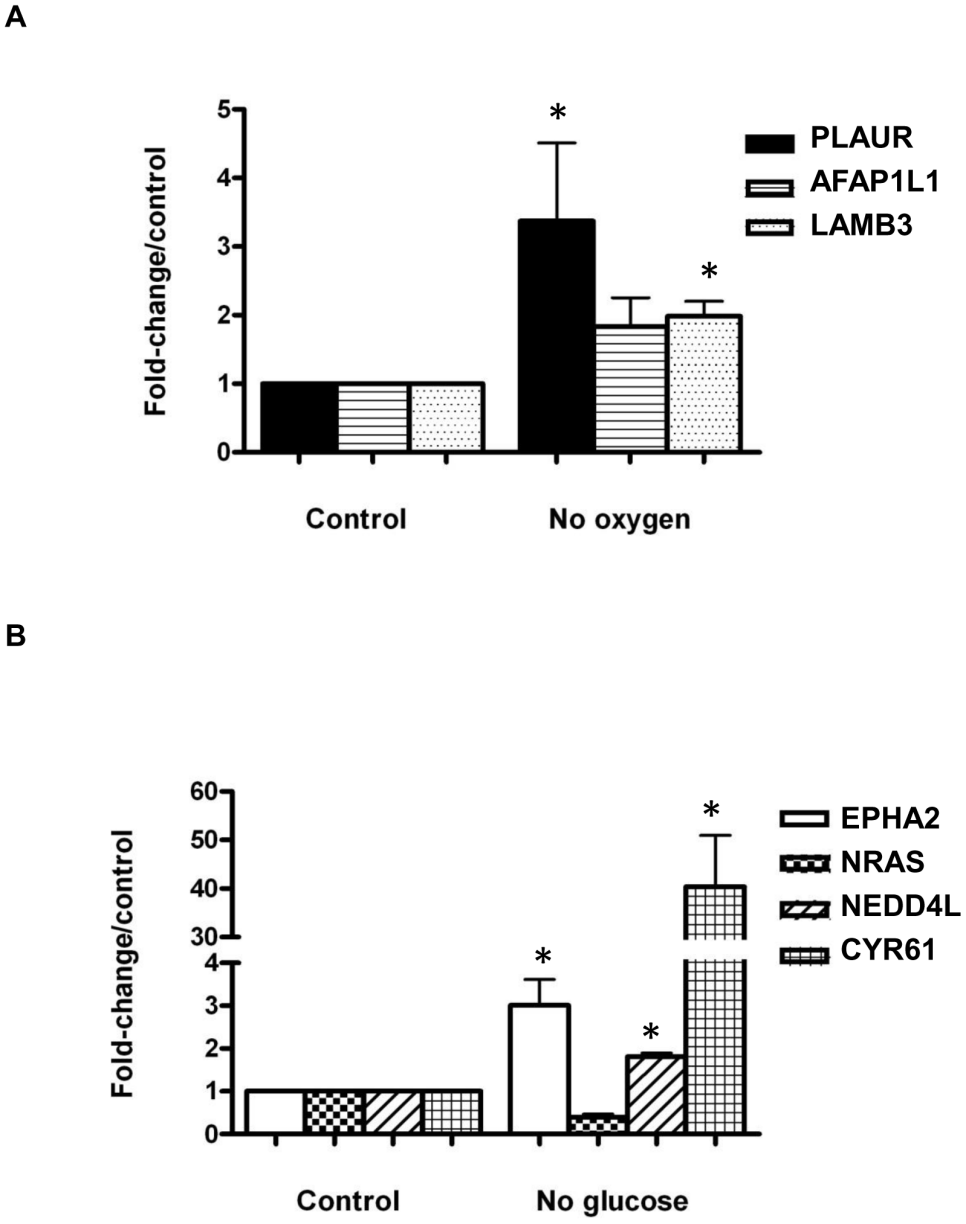
Quantitative reverse transcription-PCR to confirm expression changes in genes involved in cellular movement. cDNA levels from HCT116 cells grown in hypoxia (A) and hypoglycaemia (B) were measured for the genes of interest and normalized to β-actin, and to control. N = 5 for no oxygen and N = 3 for no glucose. * indicates significant difference relative to control; *p*≤0.05.

We used qRT-PCR to quantify expression of selected genes from both the hypoxia and hypoglycaemia groups. We choose to focus on genes involved in cellular movement identified in the cross-platform analysis as upregulated and hypomethylated. For the hypoxia group, we examined *PLAUR*, *AFAP1L1*, and *LAMB3*. Both PLAUR and LAMB3 were significantly upregulated in hypoxia, compared to control conditions (Figure 6A). The fold-changes in expression of these two genes as determined by qRT-PCR were comparable to the fold-change seen in the expression array (Table 2). Genes assessed for expression changes by qRT-PCR in hypoglycaemic conditions included *EPHA2*, *NRAS*, *NEDD4L*, and *CYR61*. All four of these genes were upregulated in the expression array analysis and hypomethylated in the promoter tiling array, except EPHA2 was not hypomethylated according to the promoter array. Significant upregulation of EPHA2, NEDD4L and CYR61 in hypoglycaemia was confirmed by qRT-PCR (Figure 6B).

### Ischemic-conditions enhance HCT116 motility

**Figure 7:**
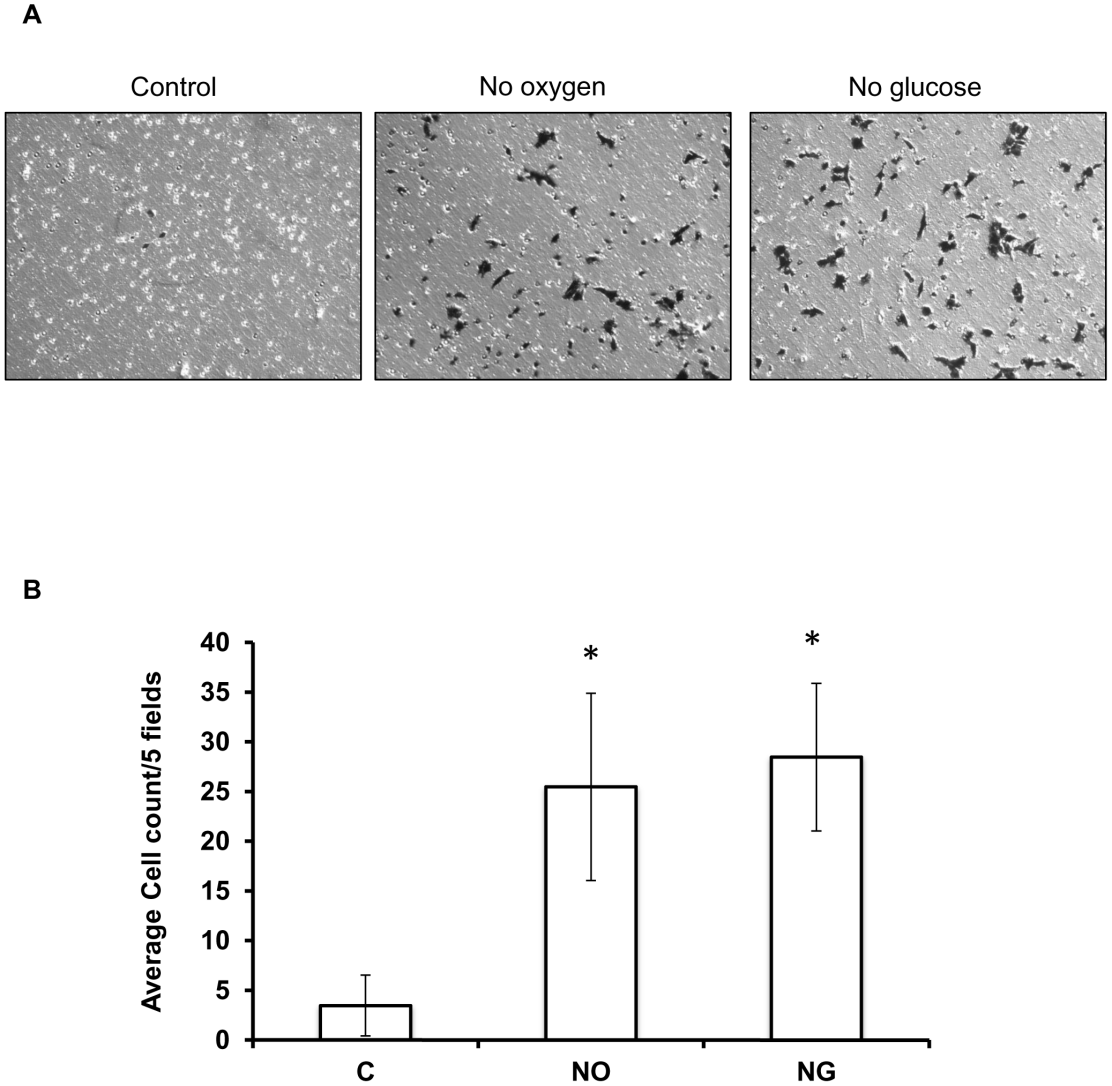
Effect of ischemia on HCT116 invasive capabilities. Cell images taken at 10× magnification from the underside of the Matrigel-coated transwells (A). Light refractive cells (black) indicate that these cells migrated through the transwell membrane. Average number of HCT116 cells which invaded Matrigel coated transwells after 48 hours exposure to no oxygen (NO) and no glucose (NG), followed by 24 hours in the transwells (B). * indicates significant difference from Control; *p*<0.01.

Invasion assays were performed to determine the effects of ischemia on cell motility. After 48 hours in ischemic conditions, it was evident that cells exposed to hypoxia and hypoglycaemia had significantly increased mobility and invasive capabilities as determined by the ability of cells to degrade and migrate through Matrigel (Figure 7). This functional assay supported our finding that elevated expression of mobility-associated genes via promoter hypomethylation in ischemic conditions translates to phenotypic changes in HCT116 cells.

## Discussion

In this study, we utilized a cross-platform approach of identifying genome-wide changes in promoter methylation and gene expression to discover how acute ischemia impacts methylation-mediated gene expression in human colorectal cancer cells. We identified and verified that genes involved in enhancing cellular movement are regulated under ischemic conditions, represented by hypoxia and hypoglycaemia *in vitro*.

Acute ischemia frequently occurs in solid tumours due to the dynamic tumour vasculature and the rapid expansion of tumours exceeding angiogenesis [2]. We previously established that acute ischemia leads to down-regulation of both cellular methylcytosine content and DNMT expression and activity [27], [28]. To pursue this finding and identify which genes are hypomethylated and upregulated by ischemia on a global scale, we used platforms that share the common Affymetrix technology base to facilitate cross-referencing of data sets. We thus used a single software bioinformatics package (Partek Genomic Suite) to import, analyze and correlate raw data from the various microarray platforms [33]. From our genome-wide, cross-platform study, we found ischemic conditions significantly upregulated genes involved in cell motility and invasion, and these genes had demethylated promoter regions identified by the arrays. Hence, our cross-platform analyses point to a set of genes that appear responsive to environmental hypoxia and hypoglycaemia via altered CpG methylation.

Previous studies have demonstrated that some HIF1-mediated genes (*e.g. CA9, EPO*) require the CpG within the hypoxia response element (HRE) to be demethylated in order for HIF1 to bind, and expression to occur [34]–[36]. S100A4, a member of the S100 family of Ca^2+^-binding proteins, is known to be involved in cancer cell motility by its ability to activate non-muscle myosin, and has been shown to be related to gastric cancer progression [34]. S100A4 was upregulated by hypoxia in ovarian cancer, with reduced methylation of the HRE in *S100A4*’s promoter regions and increased binding of HIF1 [7]. These studies provide supporting evidence towards the concept that hypomethylation is required in hypoxic conditions for gene expression to occur.

A relevant gene significantly upregulated and hypomethylated by hypoxia from our cross-platform analysis is plasminogen activator urokinase receptor, or PLAUR, the receptor for urokinase type plasminogen activator (uPA). uPA is a serine protease that catalyzes conversion of plasminogen to plasmin, which then degrades the ECM [35]. PLAUR is involved in cellular movement and metastasis [36] and expression increases during the critical transition from severe dysplastic adenoma to invasive carcinoma in colorectal cancer [35]. Additionally, PLAUR is upregulated in hypoxia [37] through HIF1 activity [38], and is responsible for hypoxia-mediated invasiveness in HCT116 cells [39]. As well, the ligand uPA is regulated by the transcription factor ETS1, and the binding sites for ETS1 in the *uPA* promoter must also be demethylated for transcription to occur [40].

Laminin beta 3 (LAMB3), a subunit of laminin-5 (laminin-332), is a basement membrane protein thought to mediate cell attachment, migration, and organization of cells during embryonic development. LAMB3 expression is higher in malignant esophageal squamous cell carcinoma than in normal tissue, and expression correlates with depth of invasion and survival [41]. LAMB3 has also been reported to be hypomethylated and upregulated in gastric cancer [42]. We confirmed that LAMB3 was upregulated in hypoxic conditions in CRC. Interestingly, one study examined colon adenocarcinoma biopsies, and saw laminin-5 positive staining was associated with budding cancer cells located at the tip of invading malignant epithelium, and that laminin-5 colocalized with PLAUR [43].

In addition to *PLAUR* and *LAMB3*, *S100A10* was another gene upregulated by both hypoxia and hypoglycaemia based on our array analysis, and has been shown to be important in cancer cell invasion and metastasis through colocalization with uPA/PLAUR system [44]. S100A10 is overexpressed in gastric cancer [45] and is essential for tumour-associated macrophage migration into tumour sites [46]. We are the first to report that *PLAUR* and *LAMB3* appear to be key gene targets in ischemia-mediated hypomethylation, similar to *S100A4*. PLAUR, LAMB3 and S100A10 could be working in conjunction with each other to increase cellular mobility in hypoxic tumours. Further studies are needed to determine if these proteins are colocalized in hypoxic tissue.

Cysteine-rich 61 (CYR61) is a member of the CCN family of growth factors. CYR61 is known to link cell surface and the ECM, and is involved in cell adhesion, proliferation and migration [47]. We have identified *CYR61* as a hypoglycaemia-responsive gene. CYR61 expression is increased in a variety of cancers including breast, melanoma, glioma, gastric, colon, bladder, prostate, and pancreas [47]. Silencing of CYR61 in osteosarcoma and esophageal squamous carcinoma cells led to reduced migration and cell invasion [48], [49]. CYR61 expression has been shown to correlate with the aggressiveness of pancreatic cancer cells, further demonstrating the role of this growth factor in metastasis [47]. To date, there are no studies showing that *CYR61* expression is regulated by promoter methylation. However, CYR61 expression is regulated by the transcription factor STAT3 [50], and others have demonstrated that the CpG in the STAT3 binding site requires demethylation for STAT3 to bind and express its target genes [51].

Two other genes that were upregulated in our analysis by hypoglycaemia were *EPHA2* and *NEDD4L*. EPHA2 is a transmembrane receptor tyrosine kinase, which is overexpressed in many carcinomas, including early stage colorectal cancer [52], and its expression is highly correlated to tumour invasion and metastasis [53]. NEDD4L is an E3 ubiquitin-ligase shown to have increased expression with gallbladder cancer progression. Using siRNA, silencing of NEDD4L led to decreased Matrigel and collagen invasion of gallbladder cancer cells, and its role in invasion is possibly due to its association with MMPs [54]. Presently, there are no studies demonstrating that promoter demethylation could be responsible for upregulation of either of these two genes.

Melanoma-associated antigens (MAGEs) are a group of genes whose expression is silenced in most normal somatic tissue, except in the testis, but upregulated in many cancers [22]. One study showed that the promoter region of *MAGE-A1* contains two ETS binding sites which must be demethylated for the transcription factor to bind and expression to occur [55]. Demethylation and expression were observed in several different cancer cell lines, and treatment with 5-aza-dC increased MAGE-A1 expression in normal fibroblasts due to the demethylation in the promoter region. Expression of MAGE-A1 and -A4 has been shown to be correlated with disease stage in melanoma patients [23]. Both demethylation and expression of MAGE-A1 and –A3 has been seen in colorectal cancer [22] and in non-small cell lung cancer patients, where expression correlated with poor prognosis [24]. Our array data indicated that two MAGE genes were demethylated by hypoxia (*MAGEA11*) and hypoglycaemia (*MAGEB1*), though no induction in expression was observed. Nonetheless, the MAGEs are a group of genes which support the importance of demethylated CpG residues in transcription factor binding sites, and that demethylation in cancer can be gene-specific.

Two other genes involved in cellular movement, *AFAP1L1* and *NRAS*, which were determined to be significantly upregulated in hypoxia and hypoglycaemia by the microarrays, respectively, were also quantified by qRT-PCR. Both of these genes did not demonstrate increased expression when checked by qRT-PCR, possibly due to cross-hybridization of non-specific sequences or splice variants, two common reasons for discrepancies between microarray findings and qRT-PCR [56].

There is sufficient evidence in the literature supporting the concept that CpGs within different transcription factor binding sites (such as HRE, STAT3 and ETS) must be demethylated in order for transcription to occur, and more importantly, some of these genes are involved in enhancing cell motility. In this study, we have contributed to this important concept by providing evidence through cross-platform array analysis for other cellular movement genes that are both hypomethylated and upregulated by ischemia. We validated changes in expression of the cellular movement genes PLAUR, LAMB3, EPHA2, NEDD4L, and CYR61 in ischemic conditions. It is well known that HIF1α levels are higher in breast and colon cancer metastases [3]. This observation complements our finding that hypoxia decreases methylation, and that a decrease in methylation in the CpG of HREs could then facilitate HIF binding and promoter gene expression. Perhaps hypoxia-induced decrease of DNMT levels is an early event in primary tumours. Cells with decreased DNA methylation are thus then primed for transcription factor binding to gene promoters which will enhance cellular movement, such as *PLAUR*. The importance of hypoxia in cancer progression is well demonstrated in cervical cancer patients whose tumours are hypoxic and have increased incidence of metastasis as compared to patients with better oxygenated tumours [57]. It is clear that hypoxia influences cancer progression, and global changes in DNA methylation are a common occurrence influencing cancer progression [58].

IPA analysis of the cross-platform data for genes that were both hypermethylated and downregulated identified genes involved in cellular functions such as cellular movement and cell-to-cell signaling and interaction (Tables II and III in Information S1). Downregulation of genes involved in cellular adhesion could conceptually contribute to increased cellular motility and metastasis. With further literature review, very few of these hypermethylated and downregulated genes are associated with cell-cell adhesion. Although an interesting idea, our data does not support suppression of cell-to-cell adhesion molecule expression as a likely mechanism responsible for the increased motility seen in ischemia.

DNMT inhibitors such as decitabine have been used clinically with some success in treating hematological malignancies such as acute myeloid leukemia [59]. However, the efficacy of these inhibitors has not been replicated in solid tumours [60]. Cancer genomes undergo hypomethylation concurrently with hypermethylation [61], thus further hypomethylating with DNMT inhibitors only resolves one half of the methylation disarray. Additionally, solid tumours have a unique feature lacking in hematological cancers: a microenvironment with ischemic regions. As we have previously demonstrated, DNMT expression and activity are reduced in ischemic conditions [28]. Therefore, using DNMT inhibitors in tumours with ischemic regions may be inefficient in cancer therapy if these regions are already undergoing hypomethylation as a result of ischemia-mediated DNMT repression.

## Conclusions

It is well known that ischemic conditions drive cancer metastasis, and here we suggest a potential mechanism for this common occurrence: through DNA hypomethylation facilitated by ischemia-mediated down-regulation of DNMTs. A significant proportion of the genes impacted by this selection pressure are involved in cell movement. This is the first report to our knowledge that provides an explanation for the increased metastatic potential seen in ischemic cells; *i.e.* that ischemia could be driving DNA hypomethylation and increasing expression of cellular movement genes.

## Methods

### Cell Culture and Ischemia Conditions

Human colorectal cancer cells, HCT116 (obtained from the ATCC), were cultured in DMEM supplemented with 10% fetal bovine serum (FBS), 50 µg/ml gentamicin, and 1 mM sodium pyruvate. Cells were grown at 37°C in a humidified chamber with 5% CO_2_. Hypoxic/anoxic conditions were generated using a Modular Incubator Chamber with continuous flushing with a humidified mixture of 95% N_2_ and 5% CO_2_. Hypoglycaemic conditions were mimicked with the use of glucose-free and pyruvate-free DMEM. Confluent monolayers of cells were trypsinized, and 3×10^6^ cells were plated onto 10 cm plates and left in standard culture conditions overnight. The following day cells were washed with PBS and switched to low serum media: DMEM and 2% FBS. 24 hours thereafter, cells were washed with PBS again and assigned to three groups: control, hypoxic, and hypoglycaemic. Cells were incubated for 48 h, followed by DNA and RNA isolation.

### Microarray Preparation and Analysis

DNA and RNA were isolated from cells as described below, and sent to the London Regional Genomics Centre, London, ON, where samples were biotin-labeled, and all microarray hybridizations, staining, washing, scanning, and data analyses were performed.

All microarray data have been submitted to the GEO database (http://www.ncbi.nih.gov/geo) under accession number GSE58233.

### Copy Number Analysis

The Affymetrix SNP 6.0 array was used to determine genomic integrity and detect drift/copy number variation in the HCT116 cells. Four µg of genomic DNA was Biotin-labelled, fragmented, and hybridized to the array. This array contains probes used in SNP analysis, as well as probes specific for CNV detection. “.CEL” files produced by GCOS software for each array were then imported into Partek Genomic Suite and analyzed using the genomic segmentation algorithm in the Copy Number Analysis workflow using the default settings, except that both CNV and SNP probes were used. All CEL files were background corrected using the Robust Multichip Average Algorithm (RMA) and results were corrected for probe GC content and fragment length. Genetic drift and Copy Number Variation in the HCT116 cells was determined by comparing the microarray data obtained to a reference data set available from Affymetrix, containing 270 mixed population samples from the International HapMap Project [29]. This array hybridization was performed in duplicate with two independent samples.

### Promoter Methylation Array

To analyze promoter methylation changes following ischemic exposure, DNA was extracted, fragmented, and enriched for 5-methylcytosine (5-mC) with the antibody-based Methylamp Methylated DNA Capture kit. Proper fragmentation of the DNA was confirmed following sonication by running the DNA on an agarose gel and observing a smear from about 800 to 200 bp. The Sigma Genomeplex Complete Whole Genome Amplification (WGA) kit was used to amplify the 5-mC enriched DNA using the manufacturer’s instructions, except that 75 µM dUTP was added to the reaction and number of cycles was increased from 14 to 20. The amplified DNA was then purified with the QiaQuick PCR purification kit. A total of 7.5 µg of the purified 5-mC enriched DNA was then hybridized to the Affymetrix 1.0R tiling array. Statistical parameters were set at a significance value of *p*<0.01 (ANOVA) over a region of at least 10 adjacent probes, and minimum length of 400 base pairs to detect significant/enriched regions in three biological repetitions. Enriched regions in treated samples versus control were determined using the Model-Based analysis of Tiling arrays (MAT) algorithm with a positive score representing a hypermethylated region, and negative score for hypomethylated region [62]. The annotations for the HG U133 Plus 2.0 array were used to determine which probe sets were associated with regions appearing to be significantly hypermethylated or hypomethylated in control versus no oxygen and control versus no glucose, and these probe set IDs were used in cross platform analyses. This assay was performed in triplicate with three independent samples.

### Genome-wide Expression Analysis

The Affymetrix HG U133 Plus 2.0 microarray was used to analyze expression changes in control versus no oxygen and control versus no glucose. Total RNA was isolated using TRIPure according to manufacturer’s instructions. RNA integrity was verified with the Bioanalyzer, with all samples having a RIN 9.3 or greater. 10 µg of RNA was used to produce Biotin-labeled cRNA, which was hybridized to the arrays. Array washing, scanning and probe quantification were carried out as per the manufacturer’s instructions using GCOS software, except that the target intensity was set to 150. For each array, GCOS output was imported as “.CEL” files into Partek Genomic Suite software, and data were normalized using the RMA algorithm as above. Probe sets significantly different in no glucose or no oxygen relative to control were determined using ANOVA, with the nominal alpha level set to less than 0.05, and then filtered for probe sets changing 2-fold and greater. In addition, for the no glucose versus control the false discovery rate (FDR) was controlled using the Benjamini and Hochberg multiple test correction. This assay was performed in triplicate.

### Cross-Platform Analysis

As per published protocols [33], Partek Software Suite was used to overlay the promoter methylation and expression data. Probe set IDs from the promoter and expression microarray platforms were imported as separate lists into Partek and compared using the Venn Analysis tool. Probe sets appearing hypomethylated and increased in expression were compared in one analysis, and probe sets appearing hypermethylated and decreased in expression in another. Lists of overlapping probe sets were then generated, and filtered in EXCEL to determine the number of unique genes represented. Proportionate Venn diagrams were then created based on these data sets.

### Ingenuity Pathways Analysis

The Ingenuity Pathway Analysis (IPA) database was used to identify gene networks and canonical pathways of the genes which were hypomethylated and upregulated. The data set containing gene identifiers and corresponding fold changes was uploaded into the web-delivered application and each gene identifier was mapped to its corresponding gene object in the Ingenuity Pathways Knowledge Base (IPKB). The functional analysis identified the biological functions and/or diseases that were most significant to the data sets. Fisher’s exact test was performed to calculate a *P* value determining the probability that each biological function and/or disease assigned to the data set was due to chance alone. In addition, networks were generated by using IPA as graphical representations of the molecular relationships between genes and gene products. Nodes were displayed using various shapes that represent the functional class of gene products.

### qRT-PCR

Quantitative reverse transcription PCR was used to validate candidate genes from the expression array. Amplification was performed as previously described [28], with the modification of 1 µg of RNA was used for reverse transcription, and the StepOnePlus system (Applied Biosystems) was used with PerfeCTa SYBR Green FastMix. *NRAS* primer sequences were: forward 5′-TGTGATTTGCCAACAAGGAC-3′, reverse 5′-CATCTTCAACACCCTGTCTG-3′. *AFAP1L1* primer sequences were: forward 5′-CAACCTCTCCCTGTCAACTG-3′, reverse 5′-TTCATTTCCCATTCCTTGGCT-3′. *EPHA2*, *LAMB3*, *NEDD4L*, *CYR61*, and *PLAUR* primers were purchased from Qiagen; these primer sequences are proprietary. *ACTB* was used as a reference gene; forward 5′-AAGATCAAG ATCATTGCTCCTC-3′, reverse 5′-CAACTAAGTCATAGTCCGCC-3′. At least 3 biological replicates were used for analysis.

### Invasion Assay

A transwell assay was used to assess the invasive potential of cells in ischemia. Cells were first exposed to no oxygen or no glucose for 48 hours in 2% FBS media as described above. Transwells (inserts) were coated with a 1∶5 dilution of Matrigel (final protein concentration of 1.55 mg/ml) in serum free DMEM, prior to seeding 1×10^5^ ischemia-exposed cells onto the Matrigel in 0% FBS. 10% FBS was used in the bottom wells of the plate to provide chemoattractants. 24 hours post seeding in transwells, transwells were removed, washed with PBS, and fixed with 4% paraformaldehdye (PFA) for 15 minutes. Transwells were then washed with PBS and stained with 0.4% crystal violet in 10% methanol for 30 minutes. Transwells were then washed with deionized water, and the upper portion of the transwell (Matrigel and cells) was wiped away and the transwell rinsed again with water. Images of the transwells were taken at 10X with an inverted microscope. Quantification was performed by counting the number of cells in 5 fields/transwell, and invasion was expressed as the average number of cells migrated per field.

### Statistical Analysis

Statistical tests for the microarrays are discussed separately under their respective sections. For all other data, one-way ANOVA was performed. If the *p* value was less than or equal to 0.05, then the Bonferroni correction was performed on pairs of data. Each assay was replicated at least three times.

## Supporting Information

Information S1 I. Complete list of hypomethylated and upregulated genes by hypoglycaemia. II. Functional analysis by IPA software of hypermethylated and downregulated genes in hypoxia. III. Functional analysis by IPA software of hypermethylated and downregulated genes in hypoglycaemia.
(DOCX)
