# Correction: Genome-Wide Analysis in Human Colorectal Cancer Cells Reveals Ischemia-Mediated Expression of Motility Genes via DNA Hypomethylation

**DOI:** 10.1371/journal.pone.0112576

**Published:** 2014-10-30

**Authors:** The PLOS ONE Staff

The first author’s name is spelled incorrectly. The correct name is: Karolina Skowronski. The correct citation is: Skowronski K, Andrews J, Rodenhiser DI, Coomber BL (2014) Genome-Wide Analysis in Human Colorectal Cancer Cells Reveals Ischemia-Mediated Expression of Motility Genes via DNA Hypomethylation. PLoS ONE 9(7): e103243. doi:10.1371/journal.pone.0103243
