# Supplementary material for: Genome-Wide Analysis in Human Colorectal Cancer Cells Reveals Ischemia-Mediated Expression of Motility Genes via DNA Hypomethylation
Source: PLoS One. 2014 Jul 31;9(7):e103243. doi: 10.1371/journal.pone.0103243 (PMC4117527; doi:10.1371/journal.pone.0103243)
Supplement: Information S1 — I. Complete list of hypomethylated and upregulated genes by hypoglycaemia. II. Functional analysis by IPA software of hypermethylated and downregulated genes in hypoxia. III. Functional analysis by IPA software of hypermethylated and downregulated genes in hypoglycaemia. (DOCX) [file pone.0103243.s001.docx]

**Supporting Information**

**I - Complete list of hypomethylated and upregulated genes by hypoglycaemia.**

|  | Expression | | Promoter Methylation | |
| --- | --- | --- | --- | --- |
| Gene Symbol | p-value | Fold-change | p-value | MAT score |
| CYR61 | 0.0000 | 31.35 | 0.009 | -3.85 |
| ETS1 | 0.0005 | 30.72 | 0.010 | -3.77 |
| KLF4 | 0.0002 | 22.29 | 0.009 | -3.85 |
| ELL2 | 0.0001 | 11.06 | 0.002 | -5.10 |
| NEDD4L | 0.0001 | 11.03 | 0.002 | -5.05 |
| LATS2 | 0.0000 | 9.27 | 0.006 | -4.13 |
| SGMS1 | 0.0004 | 8.28 | 0.002 | -5.29 |
| INTS6 | 0.0008 | 8.22 | 0.007 | -3.97 |
| HEY1 | 0.0002 | 8.18 | 0.009 | -3.82 |
| COL12A1 | 0.0012 | 6.87 | 0.006 | -4.14 |
| PHIP | 0.0006 | 5.61 | 0.009 | -3.83 |
| ARHGEF12 | 0.0003 | 5.54 | 0.004 | -4.55 |
| EPC1 | 0.0002 | 5.47 | 0.006 | -4.12 |
| SETX | 0.0009 | 5.42 | 0.003 | -4.64 |
| C16orf52 | 0.0003 | 5.10 | 0.008 | -3.93 |
| PRDM10 | 0.0012 | 4.97 | 0.006 | -4.15 |
| DCAF10 | 0.0001 | 4.88 | 0.008 | -3.94 |
| NAB1 | 0.0021 | 4.80 | 0.003 | -4.62 |
| RYBP | 0.0009 | 4.53 | 0.004 | -4.40 |
| TMOD3 | 0.0017 | 4.27 | 0.003 | -4.58 |
| TJP1 | 0.0013 | 4.23 | 0.009 | -3.82 |
| UBQLN1 | 0.0020 | 4.19 | 0.008 | -3.95 |
| OSBPL3 | 0.0001 | 4.12 | 0.005 | -4.32 |
| NRAS | 0.0030 | 4.04 | 0.009 | -3.83 |
| GPR87 | 0.0000 | 3.91 | 0.009 | -3.79 |
| ESYT2 | 0.0002 | 3.68 | 0.010 | -3.77 |
| KLF3 | 0.0004 | 3.62 | 0.009 | -3.79 |
| SOCS5 | 0.0002 | 3.61 | 0.009 | -3.83 |
| GPBP1 | 0.0009 | 3.61 | 0.008 | -3.94 |
| SLC38A1 | 0.0006 | 3.55 | 0.004 | -4.44 |
| GKAP1 | 0.0008 | 3.51 | 0.006 | -4.18 |
| EHD4 | 0.0037 | 3.48 | 0.005 | -4.31 |
| IL6ST | 0.0003 | 3.36 | 0.008 | -3.89 |
| TXNRD1 | 0.0019 | 3.34 | 0.009 | -3.84 |
| TFPI | 0.0039 | 3.30 | 0.004 | -4.40 |
| CRY1 | 0.0009 | 3.24 | 0.007 | -4.00 |
| ITGB1 | 0.0048 | 3.22 | 0.009 | -3.84 |
| PMAIP1 | 0.0006 | 3.21 | 0.007 | -4.00 |
| ABI1 | 0.0009 | 3.18 | 0.007 | -3.97 |
| RIPK2 | 0.0014 | 3.12 | 0.007 | -4.03 |
| USP53 | 0.0010 | 3.08 | 0.009 | -3.81 |
| TACC2 | 0.0004 | 3.06 | 0.007 | -4.01 |
| PPP2R2A | 0.0010 | 3.00 | 0.006 | -4.17 |
| ARAP2 | 0.0007 | 3.00 | 0.005 | -4.32 |
| SEMA6A | 0.0013 | 2.94 | 0.003 | -4.58 |
| UBE2D3 | 0.0001 | 2.93 | 0.008 | -3.93 |
| ROD1 | 0.0035 | 2.91 | 0.006 | -4.11 |
| GCM1 | 0.0004 | 2.86 | 0.003 | -4.61 |
| HECTD1 | 0.0001 | 2.79 | 0.006 | -4.13 |
| CCNL1 | 0.0011 | 2.76 | 0.009 | -3.80 |
| PTEN | 0.0039 | 2.75 | 0.009 | -3.85 |
| RNASE1 | 0.0004 | 2.74 | 0.008 | -3.93 |
| WDR1 | 0.0002 | 2.71 | 0.008 | -3.91 |
| SMAD3 | 0.0011 | 2.70 | 0.006 | -4.15 |
| WAC | 0.0043 | 2.67 | 0.010 | -3.75 |
| ARHGEF5 | 0.0000 | 2.66 | 0.002 | -5.30 |
| IL1RAP | 0.0002 | 2.58 | 0.006 | -4.06 |
| STK40 | 0.0008 | 2.57 | 0.007 | -4.02 |
| LARP4B | 0.0001 | 2.57 | 0.006 | -4.16 |
| GOLT1B | 0.0015 | 2.56 | 0.006 | -4.19 |
| PITPNC1 | 0.0011 | 2.54 | 0.005 | -4.24 |
| CDYL | 0.0002 | 2.53 | 0.003 | -4.69 |
| PSPC1 | 0.0003 | 2.52 | 0.002 | -5.17 |
| ZFAND3 | 0.0006 | 2.50 | 0.007 | -4.00 |
| MLL3 | 0.0022 | 2.49 | 0.006 | -4.08 |
| CPEB4 | 0.0017 | 2.48 | 0.006 | -4.08 |
| SYNCRIP | 0.0006 | 2.46 | 0.006 | -4.09 |
| FGFR1OP2 | 0.0019 | 2.46 | 0.004 | -4.52 |
| ADI1 | 0.0002 | 2.44 | 0.007 | -3.98 |
| BRD4 | 0.0048 | 2.42 | 0.009 | -3.85 |
| UBFD1 | 0.0033 | 2.41 | 0.007 | -4.03 |
| SLN | 0.0006 | 2.40 | 0.006 | -4.17 |
| ATG12 | 0.0044 | 2.38 | 0.009 | -3.82 |
| CSNK2A2 | 0.0023 | 2.38 | 0.010 | -3.76 |
| CDK6 | 0.0019 | 2.36 | 0.009 | -3.80 |
| TRIO | 0.0037 | 2.34 | 0.006 | -4.11 |
| NCOA3 | 0.0009 | 2.33 | 0.010 | -3.75 |
| UBR5 | 0.0005 | 2.30 | 0.006 | -4.16 |
| WDR26 | 0.0004 | 2.25 | 0.007 | -4.03 |
| RBBP6 | 0.0003 | 2.24 | 0.005 | -4.32 |
| CSNK2A1 | 0.0006 | 2.22 | 0.005 | -4.29 |
| SEMA3C | 0.0011 | 2.22 | 0.006 | -4.13 |
| PRPF4B | 0.0045 | 2.21 | 0.009 | -3.80 |
| SMAD2 | 0.0001 | 2.21 | 0.001 | -6.09 |
| NAPG | 0.0006 | 2.20 | 0.003 | -4.73 |
| TRIM11 | 0.0008 | 2.19 | 0.010 | -3.74 |
| C20orf199 | 0.0029 | 2.16 | 0.008 | -3.95 |
| PDE8A | 0.0017 | 2.16 | 0.004 | -4.39 |
| CEP76 | 0.0045 | 2.15 | 0.005 | -4.35 |
| FBXW7 | 0.0017 | 2.14 | 0.006 | -4.12 |
| SSH2 | 0.0002 | 2.13 | 0.008 | -3.95 |
| RTN4 | 0.0012 | 2.09 | 0.005 | -4.35 |
| AP1G1 | 0.0032 | 2.07 | 0.010 | -3.76 |
| ACIN1 | 0.0029 | 2.05 | 0.004 | -4.52 |
| SLU7 | 0.0042 | 2.04 | 0.009 | -3.83 |
| FNBP1 | 0.0030 | 2.02 | 0.005 | -4.21 |

II- Functional analysis by IPA software of hypermethylated and downregulated genes in hypoxia

| **Category** | **Molecules** |
| --- | --- |
| Cancer | RALA,GNA12,SUMO1,PGGT1B,POLE3 |
| Cell Morphology | RALA,STAU2,GNA12,SFR1 |
| Cellular Assembly and Organization | RALA,STAU2,GNA12,MCM4,BUB3 |
| Cellular Development | RALA,WHSC1,TRIM27,STAU2,PGGT1B |
| Cellular Function and Maintenance | RALA,STAU2,SFR1 |
| Cellular Growth and Proliferation | CDCA7,RALA,SUMO1,PGGT1B |
| Cellular Movement | TSPAN3,WHSC1,RALA,SUMO1,GNA12,PGGT1B |
| Embryonic Development | RALA,STAU2 |
| Gene Expression | SUMO1 |
| Lipid Metabolism | NAPEPLD |
| Nervous System Development and Function | TSPAN3,RALA,GNA12,STAU2 |
| Small Molecule Biochemistry | NAPEPLD |
| Tissue Development | RALA,TRIM27,STAU2,GNA12 |
| Tumor Morphology | RALA,GNA12,PGGT1B |
| Vitamin and Mineral Metabolism | NAPEPLD |
| Respiratory Disease | RALA,PGGT1B,POLE3 |
| Cell-To-Cell Signaling and Interaction | RALA,GNA12 |
| Developmental Disorder | GNA12,FECH |
| Endocrine System Disorders | GNA12,POLE3 |
| Humoral Immune Response | GNA12 |
| Inflammatory Response | RALA,GNA12 |
| Lymphoid Tissue Structure and Development | RALA,GNA12 |
| Molecular Transport | NAPEPLD,RALA |
| Neurological Disease | GNA12 |
| Gastrointestinal Disease | GNA12,POLE3 |
| Cell Cycle | MCM4,BUB3 |
| Connective Tissue Development and Function | GNA12,PGGT1B,MCM4 |
| Endocrine System Development and Function | NAPEPLD,RALA |
| Hematological Disease | FECH,POLE3 |
| Hereditary Disorder | FECH |
| Metabolic Disease | FECH |
| Reproductive System Development and Function | WHSC1 |
| Hematological System Development and Function | CDCA7,RALA,SUMO1,GNA12 |
| Connective Tissue Disorders | SUMO1 |
| Skeletal and Muscular Disorders | SUMO1 |
| Hepatic System Disease | POLE3 |
| Post-Translational Modification | SUMO1 |
| Hematopoiesis | RALA |
| Protein Trafficking | RALA |
| Protein Degradation | SUMO1 |
| Protein Synthesis | SUMO1 |
| Cell Death and Survival | RALA,SUMO1 |
| Skeletal and Muscular System Development and Function | TRIM27 |
| Immune Cell Trafficking | GNA12 |
| Organismal Injury and Abnormalities | POLE3 |
| RNA Post-Transcriptional Modification | WDR12 |
| Reproductive System Disease | POLE3 |
| Cellular Compromise | RALA |
| Immunological Disease | POLE3 |
| Cell Signaling | SUMO1 |

III- Functional analysis by IPA software of hypermethylated and downregulated genes in hypoglycaemia

| **Category** | **Molecules** |
| --- | --- |
| Cancer | RALA,GNA12,SUMO1,PGGT1B,POLE3 |
| Cell Morphology | RALA,STAU2,GNA12,SFR1 |
| Cellular Assembly and Organization | RALA,STAU2,GNA12,MCM4,BUB3 |
| Cellular Development | RALA,WHSC1,TRIM27,STAU2,PGGT1B |
| Cellular Function and Maintenance | RALA,STAU2,SFR1 |
| Cellular Growth and Proliferation | CDCA7,RALA,SUMO1,PGGT1B |
| Cellular Movement | TSPAN3,WHSC1,RALA,SUMO1,GNA12,PGGT1B |
| Embryonic Development | RALA,STAU2 |
| Gene Expression | SUMO1 |
| Lipid Metabolism | NAPEPLD |
| Nervous System Development and Function | TSPAN3,RALA,GNA12,STAU2 |
| Small Molecule Biochemistry | NAPEPLD |
| Tissue Development | RALA,TRIM27,STAU2,GNA12 |
| Tumor Morphology | RALA,GNA12,PGGT1B |
| Vitamin and Mineral Metabolism | NAPEPLD |
| Respiratory Disease | RALA,PGGT1B,POLE3 |
| Cell-To-Cell Signaling and Interaction | RALA,GNA12 |
| Developmental Disorder | GNA12,FECH |
| Endocrine System Disorders | GNA12,POLE3 |
| Humoral Immune Response | GNA12 |
| Inflammatory Response | RALA,GNA12 |
| Lymphoid Tissue Structure and Development | RALA,GNA12 |
| Molecular Transport | NAPEPLD,RALA |
| Neurological Disease | GNA12 |
| Gastrointestinal Disease | GNA12,POLE3 |
| Cell Cycle | MCM4,BUB3 |
| Connective Tissue Development and Function | GNA12,PGGT1B,MCM4 |
| Endocrine System Development and Function | NAPEPLD,RALA |
| Hematological Disease | FECH,POLE3 |
| Hereditary Disorder | FECH |
| Metabolic Disease | FECH |
| Reproductive System Development and Function | WHSC1 |
| Hematological System Development and Function | CDCA7,RALA,SUMO1,GNA12 |
| Connective Tissue Disorders | SUMO1 |
| Skeletal and Muscular Disorders | SUMO1 |
| Hepatic System Disease | POLE3 |
| Post-Translational Modification | SUMO1 |
| Hematopoiesis | RALA |
| Protein Trafficking | RALA |
| Protein Degradation | SUMO1 |
| Protein Synthesis | SUMO1 |
| Cell Death and Survival | RALA,SUMO1 |
| Skeletal and Muscular System Development and Function | TRIM27 |
| Immune Cell Trafficking | GNA12 |
| Organismal Injury and Abnormalities | POLE3 |
| RNA Post-Transcriptional Modification | WDR12 |
| Reproductive System Disease | POLE3 |
| Cellular Compromise | RALA |
| Immunological Disease | POLE3 |
| Cell Signaling | SUMO1 |
